# Rituximab Administration in Third Trimester of Pregnancy Suppresses Neonatal B-Cell Development

**DOI:** 10.1155/2008/271363

**Published:** 2008-06-25

**Authors:** D. T. Klink, R. M. van Elburg, M. W. J. Schreurs, G. T. J. van Well

**Affiliations:** ^1^Department of Neonatology, VU University Medical Center, De Boelelaan 1117, 1018 HV Amsterdam, The Netherlands; ^2^Department of Pathology, VU University Medical Center, De Boelelaan 1117, 1018 HV Amsterdam, The Netherlands; ^3^Department of Paediatrics and Infectious Diseases, VU University Medical Center, De Boelelaan 1117, 1018 HV Amsterdam, The Netherlands; ^4^Department of Paediatrics, Maastricht University Medical Center, P. Debyelaan 25, 6229 HX Maastricht, The Netherlands

## Abstract

We describe the effect on the neonate of administration of rituximab to a woman with idiopathic thrombocytopenic purpura (ITP). Rituximab, an anti-CD20 antibody, was given weekly for 4 weeks to a woman with ITP in her third trimester of pregnancy. One month after the last rituximab administration a healthy girl was born. She had normal growth and development during the first six months. At birth, B-lymphocytes were not detectable. Rituximab levels in mother and neonate were 24000 and 6700 ng/mL, respectively. Only 7 cases of rituximab administration during pregnancy were described. No adverse events are described for fetus and neonate. We demonstrate that rituximab passes the placenta and inhibits neonatal B-lymphocyte development. However, after 6 months B-lymphocyte levels normalized and vaccination titres after 10 months were adequate. No infection-related complications occurred. Rituximab administration during pregnancy appears to be safe for the child but further studies are warranted.

## 1. INTRODUCTION

Management of a pregnant patient with immune-mediated thrombocytopenia (ITP) is similar to that of nonpregnant patients [1]. Corticosteroids are very efficient and inexpensive and commonly used for this indication [2]. Although considered generally safe, steroids have been reported to have adverse effects, including oral clefts and osteoporosis [3]. Intravenous immunoglobulins (IVIGs) are a good alternative. In case of refractory ITP in pregnant patients, a combination of corticosteroids and IVIG can be given. Similar to nonpregnant patients, a splenectomy can be considered. Remission of ITP is achieved in 70% of pregnant women after splenectomy [4]. When splenectomy is considered it should be performed in the second trimester of pregnancy. The aim of treatment of ITP in pregnancy is adequate platelet count to reduce the risk of hemorrhage during labour. Both mother and neonate are at risk for severe bleeding as low-neonatal platelet count occurs in 20–25% of the offspring of ITP patients [5]. When conventional therapy fails, treatment with third- and fourth-line medications for refractory ITP is indicated. Unfortunately, their safety during pregnancy has not been established. For example, azathioprine is associated with intrauterine growth retardation and immunosupression [6, 7], and medications such as danazol and vincristin should be avoided due to their teratogenicity [8]. In order to provide safe alternatives during pregnancy, novel treatment strategies are under investigation for the management of ITP. These innovative approaches include thrombopoietin, anti-CD40 ligand, and rituximab, a monoclonal anti-CD20 antibody [1].

Rituximab is a chimeric human and murine monoclonal antibody and targets the CD20 antigen expressed on pre-B cells and mature B-lymphocytes [9]. Rituximab-opsonized B-cells are destroyed by at least three pathways: lysis through complement-mediated cytotoxicity, opsonic phagocytosis, and natural killer cell-mediated cytotoxicity [10]. The antibody was originally developed for the treatment of B-cell lymphoma [11]. Recently, however, the use of rituximab is expanded to the treatment of different autoimmune diseases including ITP [12]. The number of (autoreactive) B-cells is diminished in patients treated with rituximab. Since rituximab is an IgG-based antibody, it may cross the placenta and then interfere with fetal and neonatal B-cell development, potentially leading to immune deficiency and increased susceptibility to infections in the neonate. However, few data are available on the use of rituximab during pregnancy.

We present a case report on the effects of rituximab on a child after exposure during third trimester pregnancy. In addition, we performed a literature search for additional data in order to gain more insight on the safety aspects of rituximab administration during pregnancy.

**Case**

A 36-year-old woman was diagnosed with ITP since 1994. At that time during pregnancy, ITP was successfully treated with corticosteroids. In 1997, a splenectomy was performed. During present pregnancy, she was initially treated with corticosteroids and IVIG, but the platelet count only rose from 16 × 10^9^/L to 27 × 10^9^/L with persisting hemorrhagic diathesis. Subsequently, she was referred to our hospital and treated for refractory ITP with 60 mg prednisone daily. This treatment was continued during pregnancy until two weeks prior to delivery. In addition, from week 30 to week 34, she also received rituximab 375 mg/m^2^ intravenously weekly for 4 consecutive weeks. Due to persisting low-platelet count, rituximab administration was ceased and IVIG administration was started for two consecutive days at 37 weeks and 6 days of gestation. At 38 weeks and 2 days of gestation and one month after the last rituximab administration, labour was induced with prostaglandins. For comfort, the mother received promethazine orally and pethidine intravenously until one hour before birth. A slightly depressed girl was born with apgar scores of 7, 8, and 8 at 1, 5, and 10 minutes, respectively. Birth weight was 3780 grams (90th–95th percentile weight for gestational age according to Kloosterman [13]), length was 46 centimetre (cm) (third percentile length for gestational age [14]), and head circumference was 36 cm (98th percentile head circumference for gestational age [14]). Physical examination revealed no abnormalities. Cord blood samples were taken for blood gas analyses and measurement of thrombocytes, lymphocyte characterization, and rituximab levels. Because of the administration of opiates intravenously prior to birth, the slightly depressed neonate was subsequently admitted to the high-care facility of our neonatology department for observation. In the next 24 hours, she quickly recovered spontaneously and no adverse events occurred. The patient was transferred to the maternity ward and discharged on the fourth day after birth. She was followed up in the outpatient clinic for pediatric infectious diseases and immunology of our hospital. Up to the age of 10 months, she developed normally, and growth was not impaired. She did not have any infectious complications. Vaccination titres were normal.

## 2. METHODS

### 2.1. Rituximab serum level measurement

The rituximab serum levels in cord blood were measured using enzyme-linked immunosorbent assay (ELISA) at Xendo Drug Development, Groningen, The Netherlands. Coefficient of variation was between 4.7% and 8.2%.

### 2.2. Flow cytometry

Lymphocyte characterization was performed at the Department of Pathology. For this purpose, whole blood was incubated in Trucount tubes with fluorochrome-conjugated monoclonal antibodies directed against lymphocyte associated markers CD3, CD4, CD8, CD16/56, CD19, CD20, CD27, and CD45 (BD Biosciences, San Jose CA). After red cell lysis, lymphocyte subsets were analyzed by multiparameter flow cytometry (FACSCalibur, BD Biosciences). B-lymphocytes were quantified as the fraction of CD19 positive cells within the CD45 positive total lymphocyte gate. B-lymphocyte maturation and memory formation were analyzed as the percentage of CD19 and CD20 positive, and CD19 and CD27 positive cells within the total lymphocyte gate, respectively.

### 2.3. Review of the literature

Two Embase.com searches were performed on May 1, 2007, with EMTree-terms: (1) (“rituximab”/exp/dd_ae (adverse drug reaction), dd_it (drug interaction), dd_to (drug toxicity) AND (“newborn”/exp OR “infant”/exp OR “fetus”/exp OR “embryo”/exp OR “pregnancy”/exp)) which resulted in 19 references; (2) (“rituximab”/exp AND “pregnancy”/exp)—54 references. Duplicate references from both searches were removed, resulting in 42 references. Titles and abstracts identified were examined to select potentially relevant studies. In addition, reference lists of identified studies and review articles were examined.

## 3. RESULTS OF REPORTED CASE AND LITERATURE SEARCH

Our literature search resulted in 7 additional case reports on rituximab use during pregnancy. The relevant data of 8 cases, including our case, are summarized in Table 1.

### 3.1. Rituximab levels and B-lymphocytes in the mother

In the majority of the cases, the indication for rituximab was the presence of a B-cell malignancy. In 3 cases, including our case, the indication was a haematological disorder which involved the immune system. Regardless of the indication, the dosage was the same in all cases with a frequency of either once a week or every 14 days. Repetition of dosage was either 4 or 6 times. In combination with nonalkylating chemotherapy (CHOP), rituximab was effective in reducing tumorload in 5 cases (Table 1). In 2 cases [15, 16] and our case, maternal B-cell numbers were determined at the day of birth and were not detectable. Decker et al. [15] reported a reconstitution of maternal B-cells 12 weeks after delivery coinciding with a large decrease in serum concentration of rituximab. At birth, the maternal rituximab levels were reported to be approximately 25 mg/L when administrated a month prior to delivery. We have found equal rituximab levels in our case. Friedrichs et al. [16] reported a lower level of 9.7 mg/L, but in this case, report rituximab was administrated around 12 weeks prior to delivery. When followed up, the rituximab levels decreased corresponding with its known half-life time [15, 16].

### 3.2. Rituximab levels and B-lymphocytes in the neonate

At birth, the neonatal rituximab level in our case was 6.7 mg/L. Two other authors reported neonatal rituximab levels at birth: Friedrichs et al., and Decker et al. reported a level of 32 mg/L and 30 mg/L, respectively. Both authors reported a decline in rituximab levels consistent with the known half-life time of rituximab.

**Figure 1:**
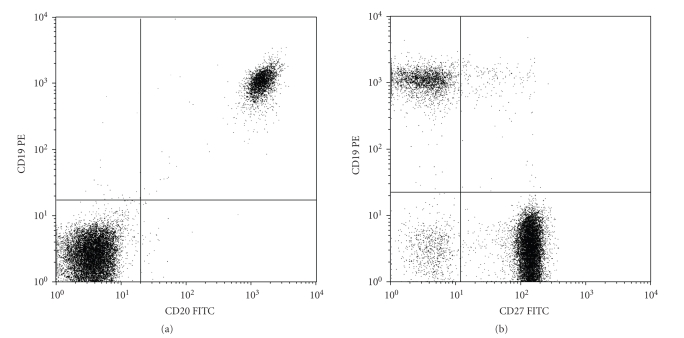
Flow cytometric analysis of peripheral blood lymphocytes, taken from the neonate at the age of six months, indicating the presence of CD19 positive B-lymphocytes (20% on average; (a) and (b)), expressing the maturation marker CD20 (99%; (a), and partly expressing the memory marker CD27 (7%; (b). Analysis was done within the CD45 positive total lymphocyte gate.

In 2 cases [15, 16] and our case, the number of neonatal B-cells at time of delivery was determined and was undetectable by flow cytometry. Time of rituximab administration during gestation did not appear to influence this outcome. In our case, the number of B-lymphocytes demonstrated a steady increase over time. Although the number of B-cells was still zero 3 weeks after birth, a rise was detected after 3 months. At the age of 6 months, the number of B-cells was in the normal range both in absolute and in relative numbers. Moreover, at this time, all peripheral B-cells were mature as indicated by CD20 positivity, and B-cell memory formation could be demonstrated by CD27 positivity (Figure 1). In addition, normal immunoglobulin (IgG) levels could be demonstrated which is consistent with findings in other case reports. In our case and in 3 other cases, [15–17] normal vaccination responses were found.

### 3.3. Clinical outcome of the neonate

During pregnancy, fetal growth and development were monitored and turned out to be normal. Indeed, in our case, the patient's birth weight was between the 90th and 95th percentile [13], within the normal range of birth weight in the Dutch population (outer limit is 97th percentile).

Five out of eight neonates were born at term age (Table 1). Of the three preterm neonates, one was delivered by caesarean section due to deterioration of the maternal condition [18]. No apparent cause for the preterm birth was reported in the other two neonates. Only one neonate [19] was described to have been treated with antibiotics for suspicion of perinatal infection. All other children did not have any complications in the neonatal period. More importantly, no single adverse event occurred.

In our case, the neonate was admitted to the high-care facility for observation due to maternal intravenously opiates administration. The arterial umbilical cord blood gas analysis was pH 7.26, CO_2_ 57.0 mmHg, 25.0 HCO_3_^−^ mmol/L, and base excess −3.1 mmol/L, indicating little intrauterine hypoxic stress. Although our patient was slightly depressed at birth, she quickly recovered. Thus, the need for further blood gas analysis was eliminated.

All the children had normal growth and development. No clinical signs of impaired immunity were observed.

## 4. DISCUSSION

Rituximab is generally considered safe for the treatment of both malignant and nonmalignant diseases. Although frequently used in women who are fertile, little is known about the safety aspects during pregnancy. Since rituximab is a chimeric antibody of the IgG isotype, it is likely to cross the placental barrier, and one should consider the risk of B-cell depletion in the child. To our knowledge, in vitro studies on placental transfer of rituximab have not been reported, but rituximab levels could be demonstrated in the exposed neonate. In our case, rituximab levels were 3.5 times lower in the neonate at birth when compared to the serum concentrations of the mother. This is in contrast with previously reported cases [15, 16] in which the rituximab levels in the cord blood were higher compared to maternal levels. As rituximab was administrated in the second trimester in these cases, time of administration during the pregnancy may contribute to this difference. Maternal IgG is transferred across the placenta by means of a specific receptor-mediated mechanism. Mother-to-fetus IgG transfer starts at week 16 of gestation. After 22 weeks of gestation, the fetal IgG levels increase rapidly [20]. Thus, when rituximab exposure starts at gestational age 16 weeks, as occurred in the two cases reported [15, 16], there has been a maximum period of opportunity for transfer. In our case, exposure was relatively short prior to birth. Although most of IgG is acquired in the last 4 weeks of pregnancy, the exposure may not have been long enough to reach the levels previously reported. Despite the lower level of rituximab in the cord blood, the number of neonatal B-lymphocytes at birth was zero. This is similar to previous reports [15, 16, 18, 19]. During follow-up, all children showed an increase of B-lymphocytes to the age-specific normal reference levels demonstrating the transient effect of rituximab (Table 1). Reconstitution of B-cells coincided with the known half-life of rituximab. Although B-cell function was initially impaired, the clinical outcome of all patients was good. None of the patients had serious infection-related complications. The explanation for this finding may be the fact that in the first few months of life, the neonate largely depends on maternal IgG for immunity, and B-cell function still has to develop. The rituximab effect may already be diminished properly before the infant depends on its own B-cell function. The data of adequate IgG and vaccination titres are supportive of this.

Although the therapeutic benefit of rituximab in malignancies is well established, its efficacy in the treatment of autoimmune diseases is still under investigation. Likewise, the exact mechanism(s) by which the B-cell depletion promoted by rituximab ameliorates autoimmune disease activity remains unclear. Despite lack of complete understanding of how rituximab targets autoimmune diseases, the antibody has been applied in the treatment of various ailments such as rheumatoid arthritis (RA), Systemic Lupus Erythematosus [21, 22], and ITP [12]. Early success with rituximab in ITP has led to its widespread use and incorporation into recent treatment schemes. A meta-analysis by Arnold et al. demonstrated [23] that rituximab was associated with a platelet count response defined as > 50 × 10^9^ cells/L. However, none of the identified studies included a control group and none met all predetermined methodological quality criteria for observational studies. The efficacy of rituximab compared with standard treatments for ITP could not be determined, and the authors strongly urge for randomized controlled trials.

In our case, rituximab administration did not result in increased platelet count and rituximab was stopped and IVIG treatment was started. This finding underlines the need for additional data to determine the effectiveness of rituximab for ITP in pregnant women.

## 5. CONCLUSION

Intrauterine exposure to rituximab appears not to be harmful to the neonate as neither in the literature nor in our case any complications related to rituximab are reported. However, data are limited to a handful of case reports, and caution should be taken when considering rituximab administration during pregnancy. In addition, rituximab should not be used indiscriminately, but should be reserved for carefully selected cases when first and second line therapy fails. Further studies are warranted to fully evaluate the safety of rituximab during pregnancy.

## Figures and Tables

**Table 1:** Summary literature search.

Mother	Herold 2001	Kimby 2004	Friedrichs 2006	Scully M 2006	Ojeda-Uribe 2006	Magloire 2006	Decker 2006	Current study
Condition	B-cell lymphoma	NH-lymphoma	Burkitt lymphoma	Thrombotic Thrombocytopenic Purpura	Autoimmune Haemolytic anaemia	Burkitt lymphoma	B-cell-NH lymphoma	ITP
Rituximab 375 mg/m^2^	Weekly 4x	Weekly 4x	Weekly 4x	Weekly 4x	—	—	Biweekly 6x	Weekly 4X
Co medication	CHOP^(1)^	—	CHOP	Plasma exchange	Corticosteroids	CHOP	CHOP	Prednisone, IgG
Administration time GA	Week 21	Week -1 to 3	Weeks 16–30	Week 27	Week 10	Week 13 to ?	Weeks 16-28	Week 30 to 34
Rituximab level D0^(2)^(ng/mL)	—	—	9750	—	—	—	25000	24000
CD19+ B-cells D0 (1 × 10^9^/L)	—	—	0	—	—	—	0	0
*Child*	—	—	—	—	—	—	—	—
GA (weeks)	35	40	41	30	38	39	33	38
Rituximab level D0 (ng/mL)	—	—	32095	—	—	—	30000	6700
CD19+ B-cells (1 × 10^9^/L) < week 1 post partum	—	0,1	0	—	0,66	—	approx 0,05	0,08
CD19+ B-cells (1 × 10^9^/L) 1 month	normal	—	—	—	1,98	—	—	0,00
CD19+ B-cells (1 × 10^9^/L) 3 months	—	—	—	—	—	—	—	0,21
CD19+ B-cells (1 × 10^9^/L) 6 months	—	—	—	—	—	—	—	2
Vaccination titres	—	Normal	Normal	—	—	—	Normal	normal
IgA; IgM; IgG (g/L) 1-2 months	—	—	—	—	0,07; 0,3; 4,5-	—	—	<0,07; 0,05; 6,1
IgA; IgM; IgG (g/L) 3 months	—	—	—	—	—	—	—	<0,07; <0,04; 2,8
IgA; IgM; IgG (g/L) ≥6 months	—	IgG normal	Normal	—	—	—	Normal	0,16; 0,53; 2,2

(1) Cyclophosphamid, doxorubicin, vincristin, prednisolon. (2) Day of birth.
